# Structural Insights into the Mechanism of Negative Regulation of Single-box High Mobility Group Proteins by the Acidic Tail Domain*

**DOI:** 10.1074/jbc.M114.591115

**Published:** 2014-09-04

**Authors:** Katherine Stott, Matthew Watson, Mark J. Bostock, Simon A. Mortensen, Andrew Travers, Klaus D. Grasser, Jean O. Thomas

**Affiliations:** From the ‡ Department of Biochemistry, University of Cambridge, 80 Tennis Court Road, Cambridge CB2 1GA, United Kingdom and; the § Department of Cell Biology and Plant Biochemistry, Biochemie-Zentrum Regensburg, University of Regensburg, Universitätsstrasse 31, 93053 Regensburg, Germany

**Keywords:** Chromatin, Intrinsically Disordered Protein, Nuclear Magnetic Resonance (NMR), Phosphorylation, Protein Kinase C (PKC), HMGB Protein, Acidic Regulatory Domain, Casein Kinase 2 (CK2), Paramagnetic Relaxation Enhancement, Protein Kinase C

## Abstract

The *Drosophila* and plant (maize) functional counterparts of the abundant vertebrate chromosomal protein HMGB1 (HMG-D and ZmHMGB1, respectively) differ from HMGB1 in having a single HMG box, as well as basic and acidic flanking regions that vary greatly in length and charge. We show that despite these variations, HMG-D and ZmHMGB1 exist in dynamic assemblies in which the basic HMG boxes and linkers associate with their intrinsically disordered, predominantly acidic, tails in a manner analogous to that observed previously for HMGB1. The DNA-binding surfaces of the boxes and linkers are occluded in “auto-inhibited” forms of the protein, which are in equilibrium with transient, more open structures that are “binding-competent.” This strongly suggests that the mechanism of auto-inhibition may be a general one. HMG-D and ZmHMGB1 differ from HMGB1 in having phosphorylation sites in their tail and linker regions. In both cases, *in vitro* phosphorylation of serine residues within the acidic tail stabilizes the assembled form, suggesting another level of regulation for interaction with DNA, chromatin, and other proteins that is not possible for the uniformly acidic (hence unphosphorylatable) tail of HMGB1.

## Teaser

**Background:** HMG-box proteins bind and bend DNA, regulated by their acidic C-terminal tails.

**Results:** The acidic tails of HMG-D (*Drosophila*) and ZmHMGB1 (maize) occlude the DNA-binding regions; the strength of the interaction is phosphorylation-dependent.

**Conclusion:** Phosphorylation provides an additional mode of negative regulation.

**Significance:** Phosphorylation of the tails of insect and plant HMG-box proteins may modulate chromatin structure and accessibility.

## Introduction

**FIGURE 1.**
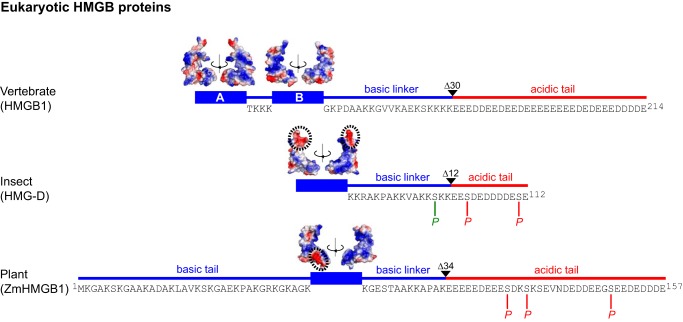
**Domain organization of eukaryotic HMGB proteins.** Sequences of linker and tail regions are shown. Truncations are indicated by Δ, and the CK2 (*red*) and PKC (*green*) phosphorylation sites are indicated by *P*. Surface electrostatics for the HMG boxes (7–9) were generated in PyMOL (10); acidic patches (see the Introduction) are encircled by *dotted lines*. The structure of the ZmHMGB1 box (residues 35–112) was derived by homology modeling (see “Experimental Procedures”).

The high mobility group (HMG)^4^ box is a DNA-binding motif that occurs in abundant chromosomal proteins such as vertebrate HMGB1 (two HMG boxes) and the functional, single-box, counterparts HMG-D in *Drosophila* and ZmHMGB1 in *Zea mays*, which bind DNA with little or no sequence specificity. The HMG box is also found in several sequence-specific transcription factors, which otherwise bear little resemblance to the non-sequence-specific HMGB proteins (1–6). The domain structure of the various abundant HMG box proteins is shown in Fig. 1 (7–10). The HMG box of *Drosophila* HMG-D is closely related to the HMGB1 B-box (40% sequence identity, 60% similarity; high structural homology), but the box has an acidic patch, and the acidic tail is markedly shorter (12 residues, including 2 serine residues, *versus* 30 consecutive acidic residues in HMGB1). The HMG box of ZmHMGB1, typical of plant chromosomal HMGB proteins, is even closer in sequence to the HMGB1 B-box than is HMG-D (48% identical, 61% similar), but also has an acidic patch (helix I), both N-terminal and C-terminal basic flanking regions, and an acidic C-terminal tail (Fig. 1), which contains several non-acidic residues (lysine, valine, asparagine, glycine, as well as serine), clustered roughly centrally in an otherwise Asp/Glu-rich region similar to the tail of HMGB1 (11).

The basic regions flanking the boxes enhance affinity for DNA (12–15), whereas the acidic tails in HMG-D (16), ZmHMGB1 (17), and HMGB1 (18) generally lower the affinity for DNA and confer selectivity for distorted DNA structures. In plant HMGB proteins, the length and positive charge of the N-terminal domain correlate with the length and negative charge of the C-terminal tail, and the two regions have been reported to interact (19).

In the structure of HMG-D bound to disulfide-cross-linked DNA (20), the basic linker appears to make contacts with the major groove via serine residues that are potentially regulated by phosphorylation; the sequence motif ^96^KKSKK^100^ is conserved from protozoans to human and is a substrate for protein kinase C (PKC) (21). The acidic tail contains two serine residues, Ser^103^ and Ser^111^, that are constitutively phosphorylated by casein kinase 2 (CK2) (22); *in vitro* dephosphorylation of these residues decreases the stability of native HMG-D and reduces the selectivity of the protein for distorted DNA substrates. The interaction between the basic and acidic tail regions of ZmHMGB1 is increased upon phosphorylation (by CK2) of serine residues in the acidic tail (23–25), and the affinity of the protein for linear DNA is decreased. In addition to the effects on DNA binding and stability, phosphorylation has also been shown to affect the distribution of HMGB proteins within the nucleus in *Arabidopsis* (26) and between the nucleus and cytoplasm in the *Chironomus* HMG box proteins (closely related to HMG-D) (27).

Given the differences between HMG box proteins that exist outside the box itself (Fig. 1), it is unclear whether, like vertebrate HMGB1 (28, 29), the single-box proteins exist in a dynamic assembly in which the HMG box associates intramolecularly with the acidic tail. In HMGB1, the DNA-binding surfaces of the two boxes and linkers are occluded in an “auto-inhibited” form of the protein, which is in equilibrium with transient, more open structures that are “binding-competent.” Although the mechanism is potentially a general one for regulation of DNA binding by acidic tails, this has not been demonstrated, and the differences illustrated in Fig. 1 may play a role. For example, the acidic patch on the HMG-D box could result in a lower affinity for its short acidic tail, and phosphorylation of the tail in HMG-D and ZmHMGB1 may play a part in modulating its intramolecular interactions.

Here we show that in both HMG-D and ZmHMGB1, the acidic tail is indeed sequestered by the HMG box and basic regions acting together. In both cases, it is the concave DNA-binding face of the box that is occluded, and the tail remains largely disordered, as for HMGB1. We also establish a role for phosphorylation of the acidic tail in fine-tuning the degree of auto-inhibition in HMG-D and ZmHMGB1; the sequence of the HMGB1 tail precludes such regulation.

## EXPERIMENTAL PROCEDURES

### 

#### 

##### Expression and Purification of ^15^N-labeled HMG-box Proteins

The plasmids encoding HMG-D Δ12 and the Ser → Cys mutants HMG-D S103C and HMG-D S111C were generated from the wild-type plasmid (pET24a HMG-D112 (16)) by QuikChange mutagenesis (Agilent). Expression of all HMG-D constructs was carried out as described (30), using ^15^NH_4_Cl as the sole nitrogen source, except that growth was continued for 12 h at 16 °C after induction.

Cell pellets were resuspended in 10 mm sodium phosphate (pH 6.0), 1 mm EDTA, 1 mm DTT, 0.5 mm PMSF, and 1 m NaCl with protease inhibitor mixture (5 μg/ml aprotinin, pepstatin A, leupeptin; 0.78 mg/ml benzamidine; and 0.5 mm PMSF). Cells were disrupted by two passes through a French press (1000 p.s.i.), and the lysate was clarified by centrifugation. Ground (NH_4_)_2_SO_4_ was added to 55% saturation (on ice, over 20 min). After a further 20 min on ice, the suspension was centrifuged, and the supernatant containing HMG-D was filtered (0.22 μm, Millipore) and then dialyzed overnight against buffer A (10 mm sodium phosphate (pH 6.0), 1 mm EDTA, 1 mm DTT, 0.5 mm PMSF). The protein was then bound to a 5-ml HiLoad SP Sepharose HP column (GE Healthcare) and eluted using a linear gradient from buffer A to 70% buffer B (buffer A plus 1 m NaCl). Fractions containing HMG-D of sufficient purity (shown by SDS/18% polyacrylamide gel electrophoresis and Coomassie Blue staining) were pooled, concentrated, and exchanged into NMR buffer (10 mm sodium phosphate (pH 6.0), 1 mm EDTA) using a Vivaspin 2 concentrator (Sartorius; 5-kDa molecular mass cut-off). Final protein concentration was estimated from the *A*_280_ using the extinction coefficient, ϵ = 19,837 m^−1^cm^−1^.

ZmHMGB1 was expressed from plasmid pT7 cm-HMGB1 (17). The sequence encoding ZmHMGB1 Δ34 (residues Met^1^–Lys^123^) was amplified from this plasmid by PCR, digested with NdeI/HindIII, and inserted into NdeI/HindIII-digested vector pT7 cm. ZmHMGB1 and ZmHMGB1 Δ34 were expressed in M9 medium supplemented with 8% ^15^N Celtone medium (Cambridge Isotope Laboratories Inc.). The proteins were purified as described previously (17).

##### Structural Model of the HMG Box of ZmHMGB1

No high-resolution structure exists in the Protein Data Bank (PDB) for the HMG box of ZmHMGB1. However, given its high sequence homology to the B box of HMGB1 (the highest match of all HMG boxes in the PDB), a model of the ZmHMGB1 box (residues 35–112) could be constructed based on entry 1HME (8) using SWISS-MODEL (31–33). The resulting model was judged to be reliable based on the QMEAN4 score (34) of 0.85.

##### NMR Spectroscopy

NMR measurements were made on ^15^N-labeled proteins (HMG-D full-length and Δ12, 1–2 mm; ZmHMGB1 Δ34, 1.2 mm; ZmHMGB1, 0.04 mm) in 10% ^2^H_2_O, 10 mm sodium phosphate (pH 7.0), 1 mm EDTA, 1 mm DTT, 0.5 mm PMSF (NMR buffer). Experiments were recorded at 30 °C (HMG-D) or 25 °C (ZmHMGB1) on Bruker DRX500, DRX600, or DRX800 spectrometers. Data were processed using the Azara suite of programs.^5^ Assignments were made using CcpNmr Analysis v. 2.1 (35). Backbone assignments were derived from three-dimensional NOESY ^15^N HSQC and TOCSY ^15^N HSQC experiments (36). Chemical shift differences were calculated using Δδ = [(Δδ^H^)^2^ + (0.15 × Δδ^N^)^2^]^½^ (37). {^1^H}^15^N heteronuclear NOE ratios (*I*_sat_/*I*_unsat_) were obtained for ^15^N-HMG-D at 500 MHz by deploying either 4 s of ^1^H saturation using a 120° pulse train or a 4-s delay prior to the first ^15^N pulse (38); errors were estimated from the standard deviation of the noise.

##### Paramagnetic Relaxation Enhancement (PRE) Measurements

^15^N-HMG-D S103C and S111C samples were incubated with 10 mm DTT at 25 °C for 15 min to ensure that the cysteine side chains were fully reduced. Spin label reagents (1-oxyl-2,2,5,5-tetramethyl-3-pyrrolidin-3-yl) methyl methanethiosulfonate (MTSL) (39) and [1-acetoxy-2,2,5,5-tetramethyl-3-pyrrolidin-3-yl] methyl methanethiosulfonate (ATSL) were added as described previously (29), and proteins exchanged into NMR buffer. Incorporation of the spin label into the protein was confirmed by electrospray ionization mass spectrometry (Dr. Len Packman, Protein and Nucleic Acid Chemistry Facility, Department of Biochemistry, University of Cambridge). PREs were extracted from the ^1^H^N^ transverse relaxation rate, Γ_2_, via a two time-point measurement: Γ_2_ = *R*_2,para_ − *R*_2,dia_ = [1/(*T*_b_ − *T*_a_)] × ln[*I*_dia_(*T*_b_) *I*_para_(*T*_a_)/*I*_dia_(*T*_a_) *I*_para_(*T*_b_)], where *T*_a_ and *T*_b_ were set to 12 μs and 8 ms, respectively (40). Errors were estimated from the noise.

##### Phosphorylation

*In vitro* phosphorylation of HMG-D was carried out by incubating 0.25 mm protein (final volume of 500 μl) at 25 °C with 5000 units of CK2 (New England Biolabs) and 1.25 mm ATP (5-fold molar excess) in 20 mm Tris-HCl (pH 7.5), 50 mm KCl, 10 mm MgCl_2_. Phosphorylation was monitored in real time by ^15^N HSQC, and by mass spectrometry (MALDI-in-source decay (MALDI-ISD) performed by Dr. Len Packman, as above). The sample was subsequently exchanged into NMR buffer for comparison with the non-phosphorylated protein.

*In vitro* phosphorylation of ^15^N-ZmHMGB1 was achieved by incubation of 40 μm protein in a total volume of 600 μl at 25 °C overnight with 0.4 μm recombinant *Z. mays* CK2 (expressed and purified as described (23)) and 400 μm ATP in CK2 buffer (25 mm Tris-HCl (pH 8.5), 10 mm MgCl_2_, 1 mm DTT) (24). The extent of phosphorylation was checked by mass spectrometry (as above) and NMR spectroscopy (after exchange into NMR buffer).

## RESULTS

Comparison of the ^15^N HSQC spectra of full-length and tail-less HMG-D and ZmHMGB1 should reveal any intra-molecular contacts of the tail with the remainder of the protein, through chemical shift perturbations, as in the case of vertebrate HMGB1 (28, 29). The contacts in HMG-D were also explored by PRE measurements, which provide a more direct measurement of distance and are less sensitive to conformational changes. The effect of tail phosphorylation was also investigated for both proteins.

### 

#### 

##### Chemical Shift Perturbation Mapping

**FIGURE 2.**
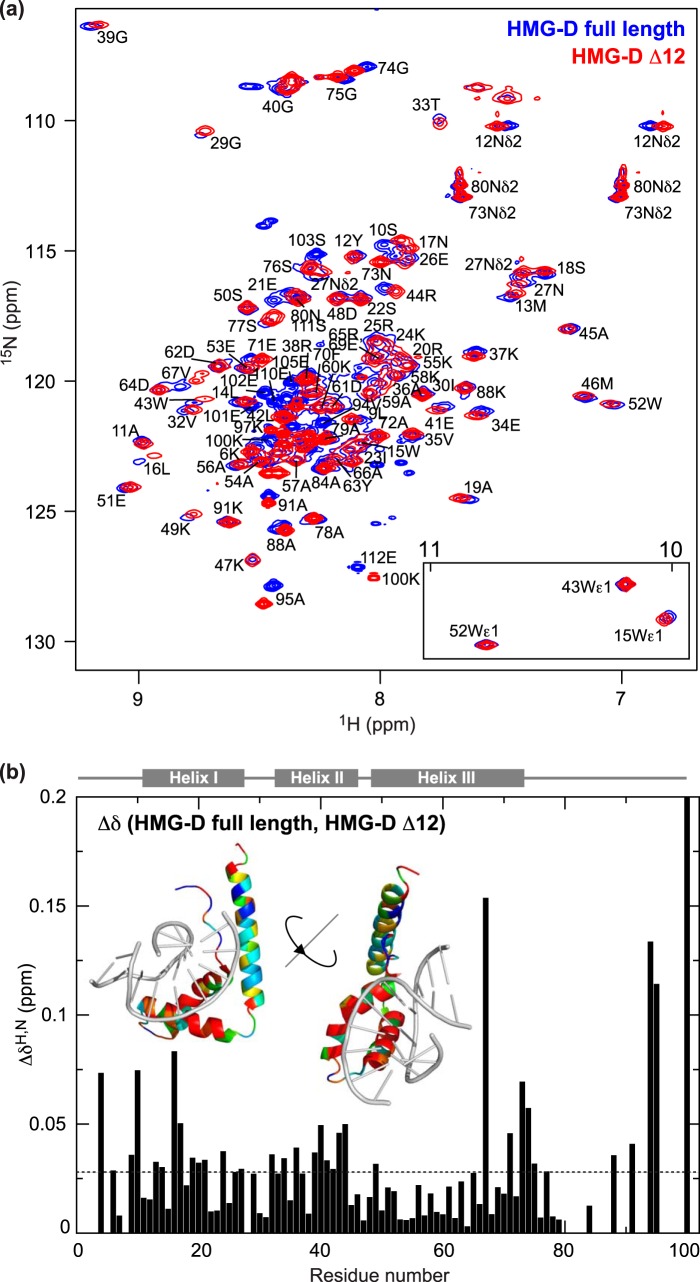
**NMR spectroscopy of HMG-D and HMG-D lacking the acidic tail (HMGD Δ12).** *a*, ^15^N HSQC spectra. *b*, chemical shift differences (Δδ = [(Δδ^H^)^2^ + (0.15 × Δδ^N^)^2^]^½^ (37)); mean Δδ 0.028 ppm (*dotted line*). *Inset* ribbon structures are based on 1QRV. Log_10_(Δδ + 1) was converted to a rainbow color ramp (*blue* = least shifted, *red* = most shifted residues); DNA is shown (*gray*) to demonstrate the similarity between the acidic-tail-binding and DNA-binding surfaces.

^15^N HSQC spectra of HMG-D are typical of HMG-box-containing proteins (28), having a combination of well dispersed peaks attributable to the HMG box and sharper, overlapping peaks in the central region, corresponding to the disordered basic linker and acidic tail (Fig. 2*a*). There is also evidence of conformational exchange on a μs-ms timescale for some peaks that are broad and weak. No evidence of intermolecular interactions was detected (^15^N HSQC spectra over a concentration range of 0.1–1 mm overlay exactly (not shown)). Chemical shift assignments were obtained using three-dimensional NOESY- and TOCSY-HSQC experiments recorded on ^15^N-labeled HMG-D and HMG-D Δ12. Of the 107 non-proline backbone ^1^H/^15^N pairs in full-length HMG-D and 95 in HMG-D Δ12, 91 and 78 pairs, respectively, could be unambiguously assigned.

Chemical shift differences between full-length HMG-D and the tail-deleted protein are small (Δδ <0.2 ppm) but widespread (Fig. 2, *a* and *b*). The mean shift difference (excluding the C-terminal residue, Lys^100^, adjacent to the deletion) is 0.028 ± 0.023 ppm (Fig. 2). This is much smaller than the value of 0.045 ± 0.062 ppm for HMGB1 (28). Nevertheless, as in the case of HMGB1, the residues that show the greatest shifts map to helices I and II, and also to a patch at the C-terminal end of helix III (in particular residue Lys^67^) and the adjacent portion of the β-strand; most of these residues lie on the concave DNA-binding face of the protein (Fig. 2*b*).

**FIGURE 3.**
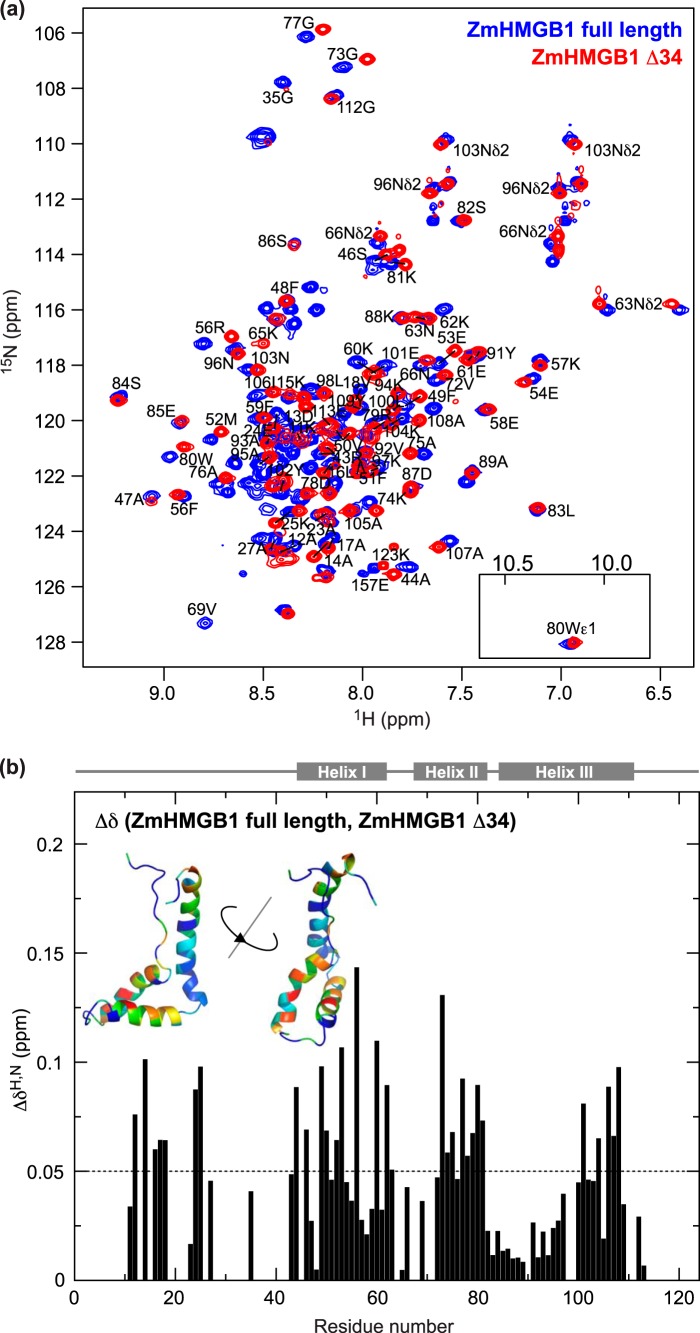
**NMR spectroscopy of ZmHMGB1 and ZmHMGB1 lacking the acidic tail (ZmHMGB1 Δ34).** *a*, ^15^N HSQC spectra. *b*, chemical shift differences (Δδ = [(Δδ^H^)^2^ + (0.15 × Δδ^N^)^2^]^½^ (37)); mean Δδ 0.050 ppm (*dotted line*). *Inset* ribbon structures are based on the homology model (see “Experimental Procedures”). Log_10_(Δδ + 1) was converted to a rainbow color ramp (*blue* = least shifted, *red* = most shifted residues).

For ZmHMGB1 (Fig. 3), peak overlap and broadening due to intermediate exchange on the chemical shift timescale precludes a complete assignment. This is most problematic in the disordered tail and linker regions where inter-residue NOE information is both sparse and ambiguous. However, a reasonable distribution of representative assignments was obtained for residues 1–123 encompassing the N-terminal tail, HMG box, and basic linker; 72 and 78, respectively, out of a possible 115 non-proline backbone ^1^H/^15^N pairs could be unambiguously assigned for ZmHMGB1 and ZmHMGB1 Δ34. Only four out of a possible 34 backbone ^1^H/^15^N pairs could be assigned unambiguously in the acidic C-terminal tail, due to the extensive overlap of the 25 Asp and Glu residues in this disordered and sequence-repetitive region. Chemical shift differences between the full-length and tail-less proteins (Fig. 3, *a* and *b*; mean Δδ 0.050 ± 0.033 ppm) are more pronounced than for HMG-D and closer to the values seen in HMGB1, and again they map primarily to the concave DNA-binding face of the HMG box (Fig. 3*b*). Residues that could be assigned in the basic N-terminal tail shift to a similar extent to those in the HMG box and the shifts are well distributed over the length of the tail (Fig. 3*b*). It may be significant that the relative mean changes in chemical shift for HMG-D, ZmHMGB1, and HMGB1 (0.028, 0.050, and 0.045 ppm, respectively) increase with increasing tail length (12, 34, and 30 residues, respectively), presumably reflecting increasing affinity of the tails.

##### Paramagnetic Relaxation Enhancement

**FIGURE 4.**
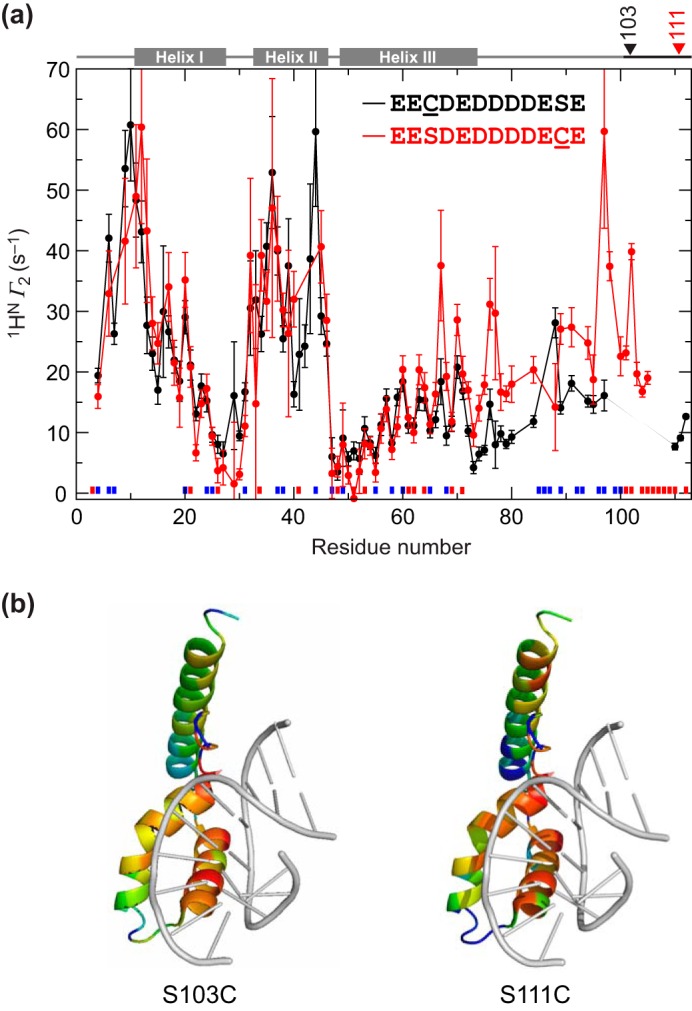
**Paramagnetic relaxation enhancement study of HMG-D Ser → Cys mutants.** *a*, PRE values measured as ^1^H^N^ Γ_2_; HMG-D S103C is in *black*, and S111C is in *red. Red* and *blue blocks* above the *x* axis indicate the positions of acidic and basic residues, respectively. *b*, ribbon structures are based on 1QRV. Log_10_(Γ_2_) was converted to a rainbow color ramp (*blue* = lowest, *red* = highest) encompassing both the S103C and the S111C datasets; DNA is shown (*gray*) to demonstrate the similarity between the acidic-tail-binding and DNA-binding surfaces.

To map contacts made by the (short) acidic tail of HMG-D using PREs, two serine-to-cysteine mutants (HMG-D S103C and S111C) were generated to permit attachment of paramagnetic probes near the distal and proximal ends of the tail (residues 101–112). The proteins were labeled with either the nitroxide-containing MTSL or its diamagnetic analog, ATSL, as carried out previously with HMGB1 (29). PREs were then quantified as the amide proton transverse paramagnetic relaxation rate enhancement (^1^H^N^ Γ_2_ = *R*_2,para_ − *R*_2,dia_) (40) for each mutant (Fig. 4*a*). Pronounced reductions in Γ_2_, indicative of proximity to the paramagnetic tag, are seen for a similar set of residues as in the chemical shift mapping (Fig. 4*b*), the main difference being a less dramatic effect seen by PRE at the C terminus of helix III, in particular for Lys^67^. One explanation might be that the changes seen by chemical shift mapping are due in part to a conformational change away from the binding site rather than a direct interaction with the acidic tail (similar chemical shift changes are seen for the B box of HMGB1 where C-terminal truncation destabilizes the last turn of the helix (41)). Another might be that the acidic tail competes for the basic linker with the acidic patch at the C terminus of helix III (Fig. 1; not present in HMGB1 or ZmHMGB1), and thus the changes seen actually correspond to detachment of the basic linker. The tail appears to make contact with both the basic linker and the HMG box.

##### Phosphorylation

**FIGURE 5.**
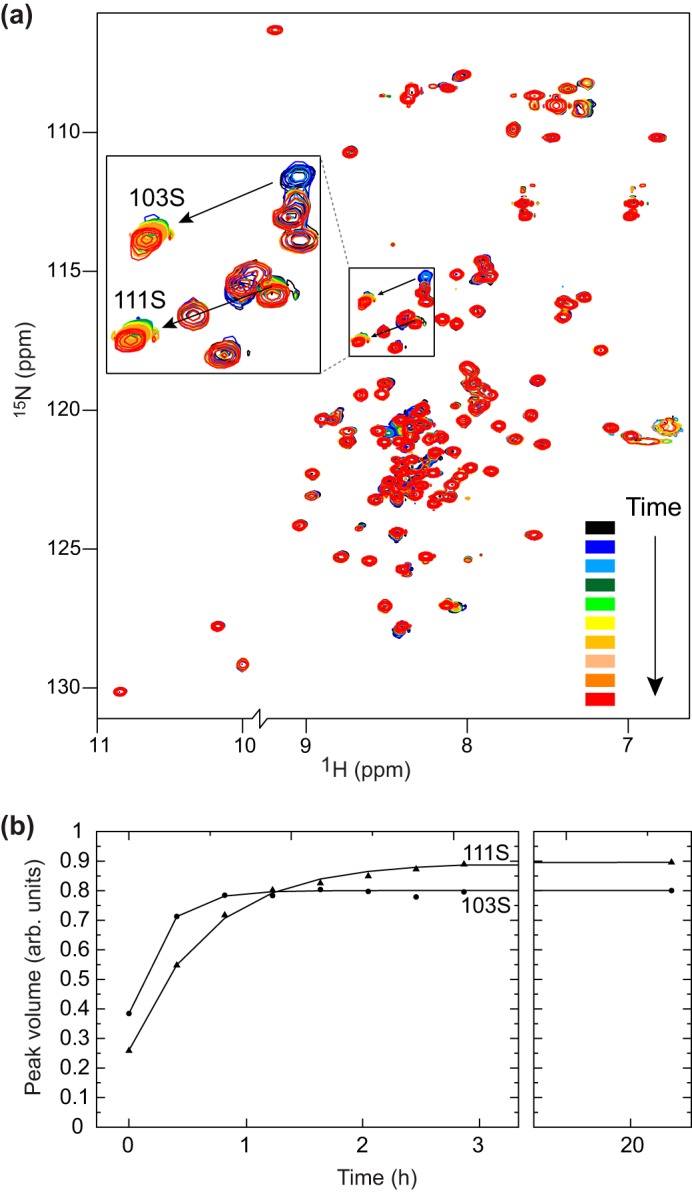
**Real-time phosphorylation of ^15^N-labeled HMG-D monitored by ^15^N HSQC NMR spectroscopy.** *a*, spectra are colored *black* through to *red* to indicate the increasing time. Peaks arising from Ser^103^ and Ser^111^ undergo a significant chemical shift change shown by the *arrow*, indicative of phosphorylation. The intensity of the original peak falls with time, whereas the intensity of the corresponding peak in the new position grows. *b*, quantification of the intensity of the new peaks seen in *a. arb. units*, arbitrary units.

Two constitutive HMG-D phosphorylation sites have been detected *in vivo* and inferred to be Ser^103^ and Ser^111^ due to their location in CK2 recognition motifs (22). To investigate the effect of phosphorylation on HMG-D, and any structural impact, ^15^N-labeled protein was phosphorylated using CK2. The reaction was followed in real time by NMR (Fig. 5). Phosphorylation was inferred to occur at both Ser^103^ and Ser^111^ as the intensity of peaks corresponding to these residues dropped over time, and corresponding peaks appeared in markedly different positions (Fig. 5*a*). This was shown directly by mass spectrometry (mass increases of 80 Da at serines 103 and 111 in sequential fragment analysis by MALDI-in-source decay (not shown)). Phosphorylation was essentially complete after 3 h, as judged by the absence of further spectral changes. Ser^103^ was phosphorylated more rapidly than Ser^111^ (Fig. 5*b*), probably due to its location in a complete CK2 recognition motif **S***XX*(D/E) (^103^**S**DED) as compared with ^111^**S**E at the C terminus (42).

**FIGURE 6.**
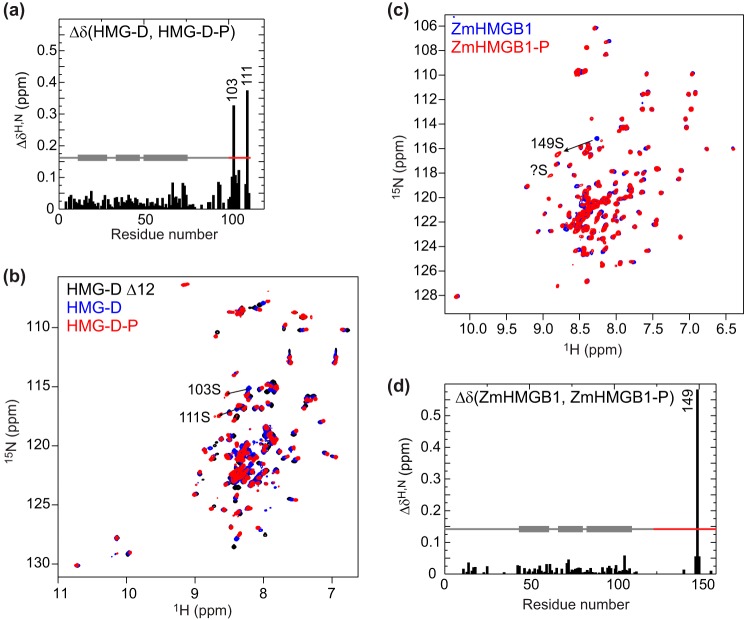
**^15^N HSQC spectra of single-box HMG proteins in the absence and presence of phosphorylation.** *a*, chemical shift differences (Δδ = [(Δδ^H^)^2^ + (0.15 × Δδ^N^)^2^]^½^ (37)) between HMG-D and the phosphorylated form, HMG-D-P; Ser^103^ and Ser^111^ are phosphorylated (Fig. 5). *b*, ^15^N HSQC spectra of HMG-D (*blue*), HMG-D-P (*red*), and HMG-D Δ12 (*black*) for comparison of shift trajectories. *c*, ZmHMGB1 (*blue*) and the phosphorylated form, ZmHMGB1-P (*red*); Ser^149^ undergoes a significant chemical shift change indicative of phosphorylation, as does another peak that cannot be assigned due to peak overlap in the spectrum of the unphosphorylated protein. *d*, chemical shift differences as in *a*.

Peaks from residues adjacent to the phosphorylation sites also shifted slightly, along with residues throughout the HMG box (mean Δδ 0.026 ± 0.018 ppm, similar in magnitude to 0.028 ± 0.023 ppm seen for tail deletion; Fig. 6*a*). Strikingly, the trajectory of the shifts was almost uniformly in the opposite direction to that following tail deletion (Fig. 6*b*), suggesting that the effect of phosphorylation is to enhance tail binding.

ZmHMGB1 is phosphorylated at 4 residues *in vivo* (cultured maize cells and immature maize kernels). Three sites were mapped to the acidic tail and could be phosphorylated by CK2 *in vitro*; Ser^149^ was fully phosphorylated, whereas Ser^133^ and Ser^136^ were phosphorylated less efficiently (75% of the protein was phosphorylated at both sites (23, 24)). We phosphorylated ^15^N-ZmHMGB1 using *Z. mays* CK2, and although the low sample concentration made it impractical to follow the reaction in real time, comparison of the fully phosphorylated protein (denoted Zm-HMGB1-P) with the unmodified protein (Fig. 6*c*) showed a major shift in the position of the peak arising from Ser^149^; this was of a similar magnitude and along a similar trajectory to that of the serine residues in HMG-D. An unassigned serine in ZmHMGB1 also shifted, most probably one of the serines (Ser^133^ and Ser^136^) previously observed to be less efficiently phosphorylated (23), or possibly both if they have overlapping chemical shifts. As in the case of HMG-D, adjacent residues were shifted slightly, but there were also widespread small changes over much of the N-terminal tail and HMG box (Fig. 6*c*), generally in the opposite direction to that caused by tail deletion (not shown), again indicating that phosphorylation results in an enhancement of tail binding. The magnitude of the changes (mean Δδ 0.014 ± 0.010 ppm) was less than for tail deletion (0.050 ± 0.033 ppm).

Phosphorylation has less of an effect on tail interactions in ZmHMGB1, as judged by chemical shifts, than in HMG-D (mean Δδ 0.026), possibly because the density of phosphorylation sites is somewhat lower in the longer ZmHMGB1 tail (three sites in 34 residues for ZmHMGB1 *versus* two sites in 12 residues for HMG-D).

##### Backbone Dynamics

Both the basic (AK-rich) regions flanking the HMG box(es) and the acidic tails are intrinsically disordered in HMG-box proteins. Interestingly, on the evidence currently available, both appear to remain largely unstructured on binding to DNA or other proteins; the HMG-D basic linker binds to distorted (disulfide-cross-linked) DNA without induction of any regular secondary structure (20), and no induced structure was detected for the acidic tail of HMGB1 on binding to the basic tails of histones H1 (43) or H3 (44).

**FIGURE 7.**
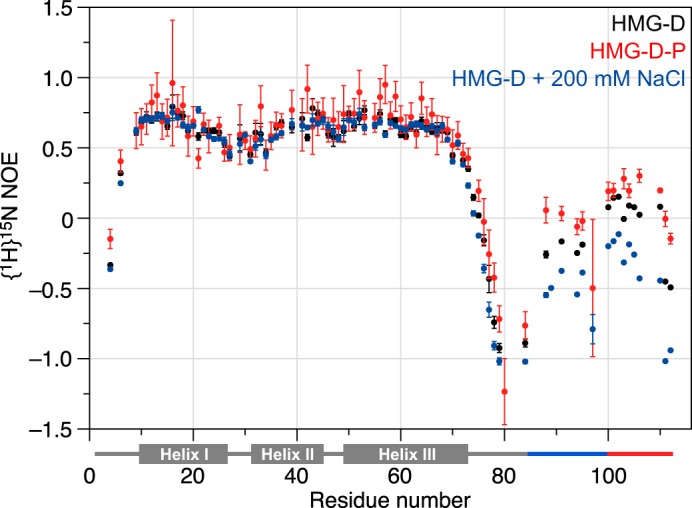
**Backbone dynamics of HMG-D (*black*), HMG-D-P (*red*) in low salt buffer, and HMG-D in 200 mm NaCl (*blue*).** {^1^H}^15^N Heteronuclear NOE values show a reduction in the extent of flexible motions in the N- and C-terminal tail regions upon phosphorylation, whereas the addition of salt results in an increase. (Note: Some *error bars* for residues in the flexible regions are not visible due to the high signal-to-noise ratio.)

Because chemical shift mapping suggests that phosphorylation enhances tail binding, we performed {^1^H}^15^N heteronuclear NOE experiments on unmodified ^15^N-HMG-D and the fully phosphorylated protein (denoted ^15^N-HMG-D-P) to establish whether phosphorylation was accompanied by a change in fast ns-ps motions in the disordered regions of the protein (Fig. 7). (This was not practical for ZmHMGB1 due to lack of available material.) ^15^N-HMG-D showed relatively high heteronuclear NOE values for the regions of known secondary structure, with a small dip corresponding to the loop region between helices I and II. Unusually, the most flexible region with large negative values was the AG-rich region preceding the basic linker, suggesting that it may be “looped out” of the assembled whole. The N- and C-terminal regions (which have {^1^H}^15^N NOE values around zero), although more flexible than the box, were less dynamic than the AG-rich loop ({^1^H}^15^N NOE ∼−1), consistent with their involvement in intramolecular interactions while retaining a degree of disorder.

Phosphorylation resulted in local decreases in the extent of motions faster than overall tumbling. The effect was small and non-uniform for the regions of established secondary structure, but significant and consistent in the disordered regions. This is very likely to be due to an increase in the affinity of the basic regions for the phosphorylated tail, which carries two extra negative charges. Similar changes in the opposite direction were seen previously for HMGB1 upon the addition of salt, when the effect of charge screening by Na^+^ and Cl^−^ ions was to weaken acidic tail interactions (29). An additional experiment was therefore performed on unphosphorylated HMG-D after the addition of 200 mm NaCl. Judging by the effect on fast motions revealed by the {^1^H}^15^N NOE, salt appeared to reduce the tail affinity by an amount similar in magnitude to the removal of the two phosphate groups.

## DISCUSSION

**FIGURE 8.**
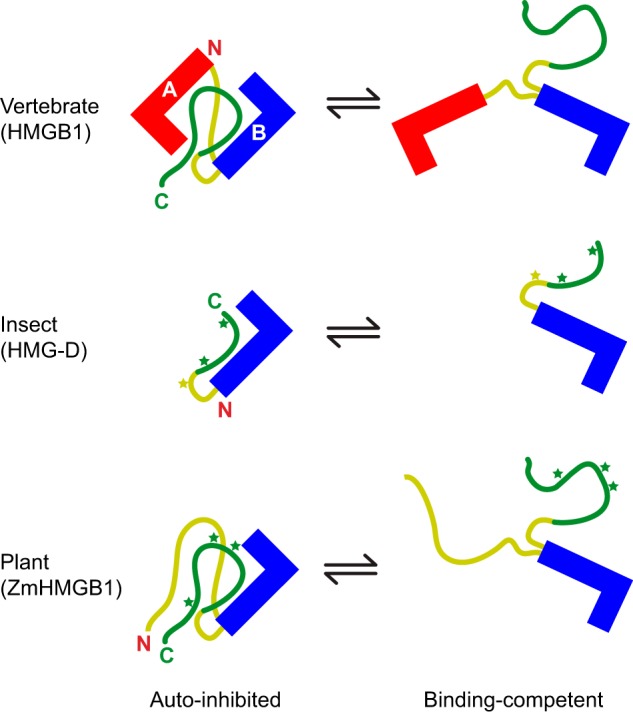
**Schematic showing the closed, auto-inhibited *versus* open, binding-competent forms of the HMGB proteins studied to date.** A-type boxes are shown in *red*, B-type are shown in *blue*, basic linker regions are shown in *yellow*, and acidic tail regions are shown in *green. Stars* indicate sites phosphorylated by CK2 (*green*) or PKC (*yellow*).

Despite the differences between the abundant HMG-box proteins from various species, the mode of auto-inhibition is conserved; the acidic tails in all cases map to an analogous interface with the HMG box(es) and basic tail/linker regions (Fig. 8). The overall effect on the structure is necessarily less dramatic for the single-box proteins than for HMGB1, where two tandem boxes are drawn together by the acidic tail to form a relatively compact, dynamic, assembly. However, in the single-box proteins, tail phosphorylation (not possible for vertebrate HMGB1) potentially adds an extra level of regulatory control.

The effect of phosphorylation on the conformation of the short acidic tails of the *Drosophila* and maize HMG-box proteins appears to involve a tightening of the existing interaction with the boxes and basic tail/linker regions. This may have consequences for protein function (*e.g.* phosphorylation disrupts the maize HMGB1-Dof2 complex, abrogating HMG-facilitated DNA binding of Dof2 (45, 46)). The local flexibility of the acidic tails is reduced on binding to the boxes and linkers/basic tail, and reduced further upon phosphorylation, but the tail retains a degree of disorder (Fig. 7), as in the case of the interaction of (phosphorylated) ubiquitin ligase with the intrinsically disordered N-terminal domain of its inhibitor Sic1 (47) and other “fuzzy complexes” (48).

For HMGB proteins, there is no detectable increase in canonical secondary structure in the free proteins on phosphorylation. However, there might be changes in structure on complex formation that are influenced by phosphorylation. For example, the basic disordered histone H1 C-terminal domain becomes structured on binding to DNA, and phosphorylation results in a decrease in the DNA-dependent α-helical content and a corresponding increase in β-structures (49).

For HMG-D, which has been well studied, the tail promotes the transient unwrapping of nucleosomal DNA (50), presumably in part through binding to the N-terminal region of H3 (44, 51); phosphorylation would be expected to influence this unwrapping and alter nucleosome stability, and consequently affect chromatin higher-order structure. HMG-D substitutes for H1 in mitotic chromosomes in the earliest stages of *Drosophila* embryogenesis (52, 53). We speculate that HMG-D phosphorylation/dephosphorylation might be key to controlling compaction/decompaction of the chromatin fiber in *Drosophila* embryos (essentially the entire population of HMG-D and its homologue HMG-Z is phosphorylated in 0–18-h embryos (22)). There might be parallels with phosphorylation of the H1 C-terminal domain in mitotic chromosomes in higher organisms (54), in marked contrast to the unphosphorylated state of linker histones in other forms of highly condensed (amitotic) chromatin of, for example, chicken erythrocyte nuclei (55, 56) and sea urchin sperm (57, 58). Exactly how phosphorylation relates to mitotic condensation remains to be established. We suggest that phosphorylation of the acidic tail of single HMG-box proteins might modulate protein function by shifting the equilibrium toward the tail-bound auto-inhibited state (29). Moreover, phosphorylation may also directly affect the structure and interaction of HMGB proteins with partners, by creating or disrupting binding motifs, providing an additional level of regulation.

## Footnotes

5 Azara, v. 2.8, copyright © 1993–2014 Wayne Boucher and Department of Biochemistry, University of Cambridge, UK.

## Footnotes

4 The abbreviations used are:

HMG: high mobility group
HMGB: high mobility group box
HSQC: heteronuclear single quantum coherence
TOCSY: total correlation spectroscopy
PRE: paramagnetic relaxation enhancement
MTSL: [1-oxyl-2,2,5,5-tetramethyl-3-pyrrolidin-3-yl] methyl methanethiosulfonate
ATSL: [1-acetoxy-2,2,5,5-tetramethyl-3-pyrrolidin-3-yl] methyl methanethiosulfonate
CK2: casein kinase 2
P: phosphorylated.
